# Peripheral Nerve Regeneration with Acellular Nerve Allografts Seeded with Amniotic Fluid-Derived Stem Cells

**DOI:** 10.1155/2022/5240204

**Published:** 2022-08-01

**Authors:** Xue Ma, Eileen Elsner, Jiaozhong Cai, Thomas L. Smith, Zhongyu Li

**Affiliations:** Department of Orthopedic Surgery, Wake Forest University School of Medicine, NC, Medical Center Boulevard, Winston-Salem, North Carolina 27157, USA

## Abstract

**Introduction:**

Tissue engineering strategies have attempted to mimic regenerating axons' environment by adding supportive types of cells other than Schwann cell to the nerve allograft. We hypothesized that allografts can be seeded with amniotic fluid-derived stem cells (AFS) to promote nerve regeneration.

**Methods:**

ANAs with AFS cells for long-gap nerve repairs were studied using a rat model. A sciatic nerve injury was created and repaired immediately with a rat acellular nerve allograft (ANA) construct alone, an ANA construct seeded with AFS cells, or with an autograft. Walking track analysis and electrophysiology were performed to document the return of motor control at 4 months post injury. Axon morphology on the nerve segments was assessed.

**Results:**

*In vivo* gait analysis showed that the ANA plus AFS cell group had significantly advanced recoveries in overlap distance, paw angle degree, paw drag, stance width, axis distance, and sciatic function index (SFI) compared with ANA alone. The ANA plus AFS cell group also demonstrated greater gastrocnemius compound muscle action potential (CMAP) ratio, sciatic axon diameter, fiber diameter, myelin thickness, *G* ratio (average axonal diameter (AD)/fiber diameter (FD)), and neuromuscular junction (NMJ) numbers compared to ANA. *Discussion*. The allograft plus AFS cell group demonstrated significantly improved functional and histological outcomes compared to allograft group alone, showing no significant difference of the nerve regeneration from the autograft group. Thus, AFS cells may be a suitable cell source to replace Schwann cells to support and accelerate peripheral nerve regeneration following large-gap nerve injury.

## 1. Introduction

Peripheral nerve injury remains a challenging clinical problem with residual functional deficits (motor and sensory) associated with attempted regeneration across irreparable nerve gaps. In addition to fibrosis in the nerve bed and at the site of injury, peripheral nerves have an inherent regenerative difficulty in overcoming gap defects [1, 2]. Although the regeneration of axons is supported by resident Schwann cells changing to a phenotype supporting growth, the environment supporting neuronal growth must establish axonal contact in a timely manner. When a nerve defect is too extensive to be repaired primarily, nerve scaffolds (e.g., conduits, allograft) and autografts have been employed with encouraging clinical results [1–4]. The use of autologous nerve grafts provides cell-rich material to promote axon regeneration. However, autograft usage is limited by donor availability, morbidity at the donor site, and nonspecific regeneration [5–7].

Acellular nerve allografts (ANAs) are promising alternative to autografts without the immunosuppressive concerns to the host tissue [8]. However, low efficacy of ANAs has been reported in nerve regeneration due to the lack of supporting cells. A recent study pointed out that limited regeneration in long acellular nerve allograft is associated with increased Schwann cell (SCs) senescence [9]. Repopulating longer ANAs requires a large amount of proliferating host SCs to promote the growth cone regeneration of the axons, which may place stress and cause eventual senescence of the SCs, leading to the failure of the efficient nerve repair. The research for the alternative of SCs has become a major trend to improve the outcomes following nerve injuries in the past decade. Numerous studies have provided evidence that cell-based enhancement of ANAs is safe and effective to repair peripheral nerve defect [10]. Stem cells that have been utilized include skin-derived stem cells, adipose-derived stem cells, and mesenchymal stem cells [7, 10]. Studies utilizing these supportive cells suggest that improvements in overcoming gap distances can be achieved in the presence of these cells [11–13]. One disadvantage that these autologous stem cells share is that they all require removal of tissue from the patient, processing of tissues, and then return of the stem cell back to the patient. Because of time constraints and regulatory impediments associated with this technology, another source of stem cells is necessary in order to provide “off the shelf” utility without additional regulatory concerns.

Amniotic fluid-derived stem (AFS) cells have multipotency to differentiate into all three embryonic germ layer cell types; they also demonstrated a lack of immunogenicity and have the potential to differentiate and take on nerve cell characteristics in the presence of biochemical cues *in vitro* [14, 15]. One of the advantages of using AFS cells for peripheral nerve regeneration is that they do not require human embryo tissue for their isolation, thus avoiding the controversies associated with human embryonic stem (ES) cell applications. In addition, AFS cells have been shown to produce angiogenic and neurogenic growth factors in their undifferentiated form [16, 17]. Hence, these cells have been theorized to have the potential to support nerve regeneration by both supplying growth factors and possibly becoming incorporated into the regenerating nerves. AFS cells have been studied recently for their abilities to augment growth of injured nerve across a nerve gap using fibrin glue or biodegradable nerve conduit [17, 18]. However, the combination of AFS cells and ANA and their synergistic effects on peripheral nerve injury and repair are still poorly understood. In this study, we evaluated the ability of acellular nerve allografts (ANA) seeded with AFS cells to promote and accelerate nerve regeneration after a 1.5 cm nerve transection defect in a rat model. To our knowledge, this is the first study to evaluate the effects of ANA seeded with AFS cells in a rat sciatic nerve transection model. We found that AFS cell supplementation to ANA (i) improved the motor functional recovery and (ii) enhanced histological outcomes in nerve and muscle compared to ANA construct alone.

## 2. Material and Methods

### 2.1. In Vitro Cell Culture and Cell Seeding on Allografts

**Figure 1:**
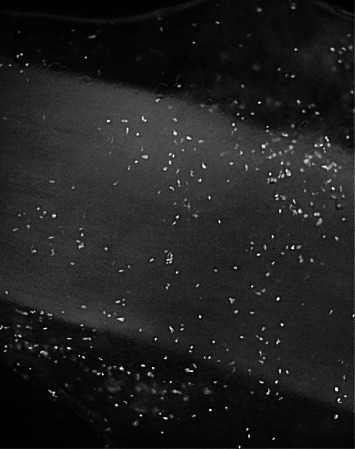
DAPI staining of longitudinal section of a sciatic nerve allograft. Magnification ×100.

Rat sciatic nerves were harvested from 3-month-old Lewis rats, and the acellular allografts (ANA) were processed by AxoGen Corp, FL. The AFS cells were a gift from NuTech, Inc, AL. The cells were cultured and expanded in modified Dulbecco's Eagle's medium (DMEM, Invitrogen) containing 20% fetal bovine serum (FBS) and 1% penicillin–streptomycin. The 1.5 cm long decellularized sciatic nerve allograft was gently perforated of the epineurium using a microneedle array. 1.5 × 10^6^ cells were suspended in 50 *μ*l media then injected underneath the epineurium of the allografts from both ends using a 26 G syringe. Seeded grafts were placed vertically at the bottom of a small centrifuge tube covered with DMEM containing 20% FBS overnight then transferred to a 48-well plate for an additional 48 hours before being implanted into the left sciatic site of the animal. After 60 hours of incubation, the allograft was fixed in 4% of paraformaldehyde for 1 hour and the AFS cell attachment to the allograft prior to implantation was assessed by DAPI staining (Invitrogen, CA) on 7 allografts (Figure 1).

### 2.2. Surgery Procedure

**Figure 2:**
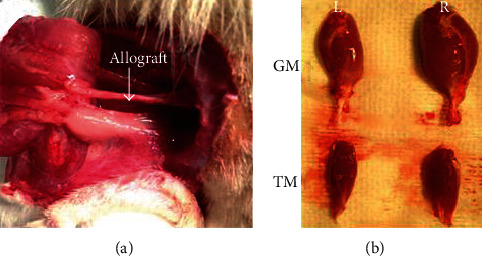
An allograft with AFS cell implant at the end of 4 months postinjury. (b) Isolated gastrocnemius (GM) and tibialis muscles (TM) from the experimental (L) and contralateral control side (R).

All animal use was approved by the Animal Care and Use Committee (ACUC) of Wake Forest University Health Sciences. Three groups (autograft, ANA, or ANA plus AFS cells), 12 male Lewis rats per group, underwent left sciatic nerve transection surgery. The rat was anesthetized using isoflurane (1.5-2.5 volume %), initially in an induction chamber. Anesthesia was maintained via a nosecone at 1.5 volume%. The posterior aspect of the left hind limb was shaved with clippers, cleansed with betadine scrub, and disinfected with betadine solution. Using aseptic technique, a posterolateral incision was made and the sciatic nerve was exposed by dissecting the muscle plane. A 1.5 cm nerve defect was created and repaired using 10-0 nylon and standard microsurgical technique by interposing a 1.5 cm nerve allograft seeded with 1.5 × 10^6^ AFS cells previously prepared or ANA construct without cells. For the autograft group, the sciatic nerve was transected and flipped then sewn back to repair the defect. Following nerve repair, the muscle was approximated using interrupted sutures of 5-0 coated Vicryl. The skin was closed by approximating the cut edges using stainless steel surgical wound clips and subdermal sutures 5-0 coated Vicryl. The rat was given buprenorphine for postsurgical analgesia (0.01 mg/kg, SC) at the end of surgery (Figure 2).

### 2.3. Walking Track Analysis

**Figure 3:**
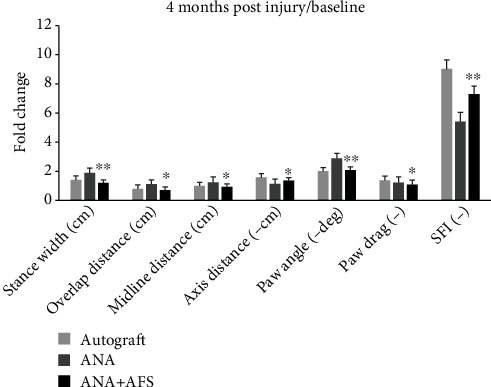
Gait indices of baseline and 4 months post nerve transection injury repaired by ANA plus AFS, ANA, and autograft group. Fold change ratio of 4 months post nerve transplantation to baseline indices demonstrated significant improved gait recovery in the ANA plus AFS compared to the ANA group alone. ^∗^*p* < 0.05; ^∗∗^*p* < 0.01.

Gait recovery is an indicator of return of motor control. The DigiGait Imaging System (Mouse Specifics Inc. MA) was used to test the motor function recovery of the allograft reconstruction following sciatic nerve defect in Lewis rats [19]. The DigiGait System imaged the underside of the running rat with a high-speed digital video camera continuously (188 frames/second) and generates digital paw prints which can be translated to dynamic gait signals of the temporal record of paw placement relative to the crystal treadmill belt. The return of motor control at 4 months after sciatic autograft, ANA, or ANA plus AFS cell implantation was documented. Each animal was compared to their preinjury walking track values. This technique permits use of the highly sensitive repeated measures analysis of variance for these animals and is capable of detecting slight differences between groups. 24 parameters at the end of 4 months following injury were analyzed (Figure 3, Table 1).

### 2.4. Electrophysiology Analysis

Cadwell EMG Sienna Wave System was used for the electrophysiology testing. 4 months after the nerve autograft, ANA, and ANA plus AFS cell implantation, rats were anesthetized with isoflurane and the regenerated and contralateral sciatic nerves were exposed. At first, tibial and peroneal branches distal to the regenerated gap were briefly stimulated to test for plantar flexion and foot eversion. After the viability of the nerve was assessed, electromyographic analysis was examined by stimulating the regenerated nerve proximally (suture sites were taken as referral points) with a monopolar cathodic electrode at 1 mA, the reference anode was placed on the rat chest. The stimulating–recording electrode distance was verified visually using a ruler. Muscle contractions were recorded by electrodes placed into the gastrocnemius muscle (medial and lateral) and tibialis muscle of both experimental and control limbs. Compound evoked muscle action potential (CMAP) was recorded by three consecutive stimulations that were averaged for CMAP delays and amplitude measurement. The measurement of CMAP was converted to ratios of the injured side to the normal contralateral side to eliminate the influence of anesthesia [17].

### 2.5. Tissue Harvesting and Histomorphometric Analysis

#### 2.5.1. Nerve

The animals were euthanized with intracardiac injection of saturated potassium chloride. The ANAs and contralateral nerves were harvested together with the proximal and distal nerve stumps. The nerve samples were fixed in 4% paraformaldehyde or 2% osmium tetroxide, dehydrated, and later embedded in paraffin or resin. Serial 5 *μ*m sections were cut 1 mm distally to the distal suture in the ANA to assess the regenerating nerve fibers penetrating to the distal nerve stump. The slides were stained with toluidine blue and examined under Zeiss light and electronic microscopes (Thornwood, NY) at 200x and 3700x final magnifications. The images were analyzed using ImageJ software to measure regenerated axons. The number of axons was counted, and the outlines of myelinated axons and total axons were manually traced. The cross-section area, the number of myelinated fibers (*n*), myelin thickness (MT), average axonal diameter (AD), and fiber diameter (FD) were assessed using ImageJ software (Wayne Rasband). The *G* ratio was calculated as AD/FD, and the fiber density calculated as number of fibers per square millimeter. The axonal area (_*π*AD/2)^2^ and the fiber area (_*π* FD/2)^2^ were obtained assuming the circularity of the nerve fiber area. The myelinated area was measured as the difference between fiber area and axonal area [20]. Axon areas were counted at minimum of 5 areas for each transverse section, and 15 sections per animal were analyzed for the different experimental groups.

### 2.6. Muscle

The gastrocnemius and tibialis muscles from both the experimental and contralateral side were harvested and weighed (Figure 2). The ratio of the experimental and contralateral muscle weights was calculated to measure the recovery of atrophy. 10 *μ*m cross sections of muscle were cut from the midline maximal area and stained with *α*-bungarotoxin at 1 : 2000 (Thermo Fisher, NY) to visualize neuromuscular junction replenishment following nerve injury and repair. 10 continuous sections of gastrocnemius and tibialis anterior muscles were analyzed per sample.

### 2.7. Statistical Analysis

Results were reported as mean values and the standard error of the mean (SEM). One-way ANOVA test with Bonferroni multiple comparisons was used to determine the statistically significant differences between experimental groups. The following conventions were used: significant, ^∗^*p* < 0.05; very significant, ^∗∗^*p* < 0.01; and extremely significant, ^∗∗∗^*p* < 0.001.

## 3. Results

### 3.1. In Vitro Cell Seeding on Allografts

Rat acellular allografts seeded with 1.5 × 10^6^ AFS cells were stained with DAPI and viewed under a confocal microscope (Zeiss Oberkochen, Germany). Seeded AFS cells were viable and spread evenly longitudinally through the nerve fibers 60 hours postinjection. The cells seeded into ANA at the end of 60 hours before implantation were 1.1 × 10^6^ ± 1203 per graft (*n* = 7) (Figure 1).

### 3.2. Walking Track Analysis

Gait analysis of 24 parameters at the end of 4 months following injury indicated that there were no significant differences in stance/swing ratio, stride time, stance factor, swing stride percentage, brake stride percentage, propel stride percentage, stance stride percentage, brake stance percentage, propel stance percentage, hind limb shared stance percentage, step angle, and stride length among three groups.

The autograft group showed significant enhanced recovery at stance width, overlap distance, ataxia coefficient, axis distance, and SFI compared to the ANA and ANA plus AFS groups. The ANA plus AFS group exhibited improved functional recovery in stance width, overlap distance, midline distance, axis distance, paw angle, and paw drag than the ANA group alone and did not show significant differences from the autograft group in these parameters, indicating beneficial regenerating ability of AFS cells at the end of 4 months following a long nerve gap injury. In addition, although the SFI of the ANA plus AFS group did not return to the level of the autograft group, the ratio of 4 months postsurgery to the baseline was significantly higher than allograft alone, suggesting an overall superior sciatic function recovery to the ANA group (^∗^*p* < 0.05 and ^∗∗^*p* < 0.01 in all indices, Figure 3; Table 1).

### 3.3. Electrophysiology Analysis

**Figure 4:**
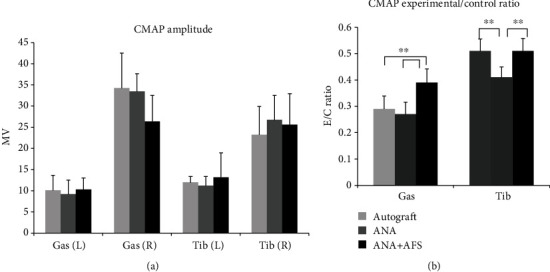
(a) Mean amplitudes of compound muscle action potential (CMAP) after stimulation of regenerating and contralateral control sciatic nerve with a monopolar electrode proximally. (b) Ratio of amplitude of experimental to contralateral CMAP of gastrocnemius and tibialis muscle in the ANA, ANA plus AFS, and autograft groups. ^∗∗^*p* < 0.01.

Electrophysiological analysis of CMAP indicated that the ANA plus AFS cells group had significant higher experimental/control ratio of wave potentials on the gastrocnemius muscle compared with the autograft and ANA groups (left CMAP (mv) autograft vs. ANA vs. ANA+AFS: 10.14 ± 3.52 vs. 9.20 ± 3.33 vs. 10.32 ± 2.7; right: 34.25 ± 8.25 vs. 33.45 ± 4.2 vs. 26.37 ± 6.17; E/C ratio: autograft vs. ANA vs. ANA+AFS: 0.29 ± 0.04 vs. 0.27 ± 0.04 vs. 0.37 ± 0.05, *p* < 0.01 between AFS+ANA and autograft, AFS+ANA, and ANA). CMAP ratio of the tibialis muscle had no significant differences between the autograft and ANA plus AFS groups but was significantly higher than the ANA group alone (left: 12.00 ± 1.39 vs. 11.20 ± 2.17 vs. 13.17 ± 5.80; right: 23.24 ± 6.69 vs. 26.75 ± 5.78 vs. 25.60 ± 7.34; E/C ratio autograft vs. ANA vs. ANA+AFS: 0.51 ± 0.04 vs. 0.41 ± 0.03 vs. 0.51 ± 0.04, *p* < 0.01 between AFS+ANA and autograft, AFS+ANA, and ANA, Figure 4).

### 3.4. Histomorphological Analysis

**Figure 5:**
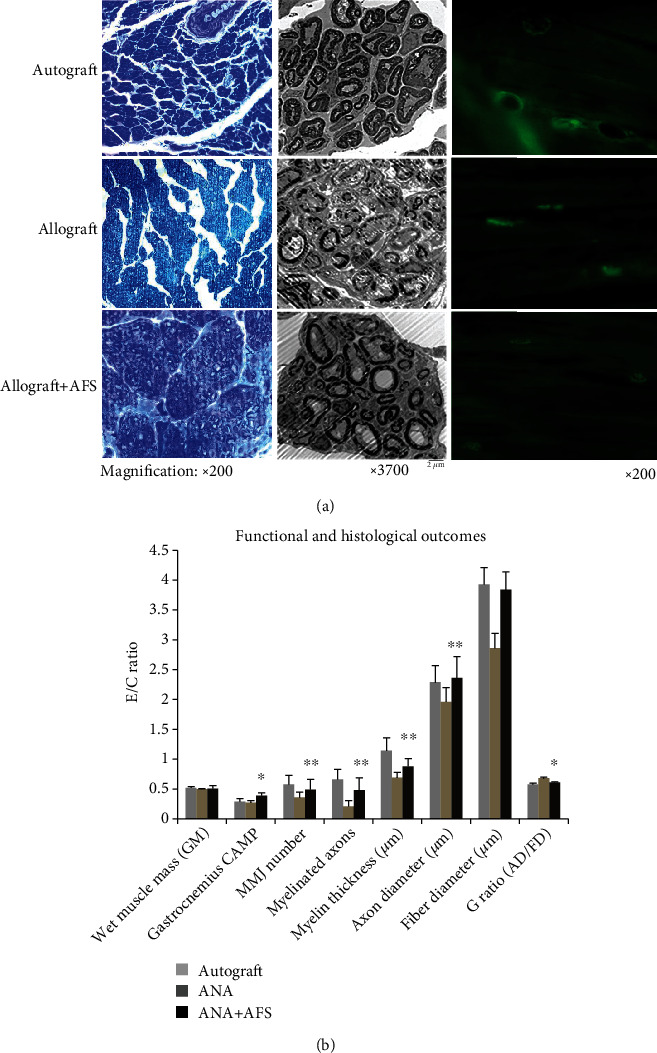
(a) Nerve histology at 4 months post-graft implantation. Toluidine blue staining of cross sections of nerve grafts 1 mm distal to the suture site (left column, ×200 magnification), electronic microscopic pictures of nerve fibers (middle column, ×3700), and immunohistochemistry staining of NMJ (right column, ×400) in 3 experimental groups. (b) Normalized experimental hind limb to contralateral control limb measurements of functional and histological outcomes in 3 experimental groups. ^∗∗^*p* < 0.01 and ^∗^*p* < 0.05, significantly different to the ANA group.

Evaluation of cross sections through the distal part of the regenerated nerves was conducted by light and electronic microscopy. The ANA plus AFS cell group demonstrated remarkably increased number of myelinated axon, axon diameter, fiber diameter, myelin thickness, and *G* ratio compared to the ANA group (Table 1, Figure 5(b)). On light microscopy, the ANA plus AFS group showed well-aligned and regenerated nerve fibers, whereas the fibers of the ANA group had an overall disrupted endoneurium architecture. On scanning electron microscopy, the ANA plus AFS group demonstrated significantly greater number of regenerated nerve fibers, axons, and myelinated axons with thicker myelin sheath. Immunohistochemistry analysis also showed that the ANA plus AFS group had significant increased number of neuromuscular junction (NMJ) with more complexed morphology, closely resembling the NMJ morphology in the autograft group (Figure 5(a)).

## 4. Discussion

In this study, we seeded AFS cells onto the ANAs and implanted the conduits to repair a 1.5 cm nerve gap for 4 months. The functional tests used to evaluate the nerve graft regeneration include treadmill walking analysis, electrophysiology, and histological analysis. The ANA plus AFS cell group showed superior axonal regeneration with enhanced motor function recovery compared with the control ANA group. AFS cell-treated animals had significantly improved performance in sciatic nerve regeneration with increased number of myelinated axon, axon diameter, fiber diameter, myelin thickness, and *G* ratio. This group also showed greater NMJ number with more complexed morphology, indicating the accelerated muscle innervation at the end of 4 months following injury. The treadmill walking analysis and electrophysiological analysis clearly demonstrated the improvement of multiple motor function parameters and CMAP, suggesting that the addition of AFS cells to ANAs had remarkably functional regenerative effects.

Pedrini et al. have conducted a thorough systematic review and meta- analysis on cell-enhanced acellular nerve allografts for peripheral nerve reconstruction. They compared various endpoints including Sciatic Functional Index (SFI), nerve conduction velocity, compound muscle action potential (CMAP) and histomorphometry. The conclusion was that ANAs supplemented by supporting cells had an overall comparable outcomes to autografts, some of the cell-enhanced ANAs showed superiority to other study groups [10]. Among the reviewed transplanted stem cells, bone marrow-derived stromal cells (BMSCs) were the most used type of stem cells, followed by adipose-derived stem cells (ASCs) and neural stem cells (NSCs).

Recently, gestational tissues such as placenta, placental membrane, and amniotic fluid have attracted wide attention in regenerative medicine as an abundant source of highly multipotent and immunosuppressive cells. The advantages of these gestational tissues are easy collection, which are usually discarded after birth or through routine amniocentesis and minimal ethical and legal concerns associated with the usage and the convenient application clinically. The amnion is the inner part of the amniotic sac that contains the fetus and amniotic fluid. The amnion is derived from ectoderm and mesoderm, and the amniotic fluid contains a mixture of stem cell types including amnion epithelial cells and amniotic fluid stem (AFS) cells that possess multipotent differentiation, anti-inflammation, and low immunogenicity characteristics [7, 9, 11, 14, 21]. The stem cells cultured from these tissues have the potential to differentiate into a variety of cell lineages including osteogenic, myogenic, neurogenic, hepatogenic, cardiac, and endothelial, which provide novel and noninvasive stem cell therapies for potential clinical applications of treating different diseases [15, 22].

It is currently unclear how human stem cell therapies contribute to peripheral nerve regeneration. A variety of different sources of stem/precursor cells are under study to determine their potential for peripheral nerve repair [14]. However, there are many unanswered questions regarding how cell transplantation therapies can be optimized for clinical use. In the present study, we showed that the supplement of AFS cells to ANA dramatically improved the functional outcomes in the *in vivo* preclinical perspective. As regards motor function assessment, the treadmill computerized gait analysis system captures the locomotion of the running animal continuously and generates the digital paw prints which can be translated to dynamic gait signals. This method allows the kinematics of gait of each limb to be analyzed precisely in each animal longitudinally, and the number of animals required for each cohort group was significantly reduced. We found that the overall gait function of injured limb in all 3 groups (12 animals per group) did not return to the extent of baseline at the end of 4 months postinjury. However, the AFS plus ANA group demonstrated improved motor functional recovery in stance width, overlap distance, midline distance, axis distance, paw angle, and paw drag parameters. Most of these indices reflect the changes of balance and postal stability affected by the lesions of sciatic nerve [23]. For instance, animals with sciatic nerve injury tend to adopt a wider stance when running on the treadmill, probably to compensate for the center of gravity shift during the movement. The application of AFS cells significantly reduced the stance width compared with the ANA group, suggesting the restoration of a more stable and smooth walking pattern.

We also found paw angle value was significantly decreased with AFS cell treatment. This factor is considered as the level of outward rotation of the paw and is usually used to evaluate the return of tibialis anterior muscle function. Our results were in agreement with other studies [23–26] that the angles of hind paws in relation to the long axis of the body were significantly different under pathologic conditions compared with normal gait. The ANA plus AFS group showed remarkably lower value of paw angle than that of the ANA group, indicating the enhanced regeneration of the perineal motor axons into the tibial nerve following sciatic nerve transection and repair with AFS cell supplement.

Sciatic function index (SFI) has been well accepted for reflection of the overall nerve function recovery after transection injury. In our *in vivo* study, all 3 groups of animal had significantly impaired SFI 1 month after nerve injury, and the SFI gradually improved over time but all of the groups did not restore to the level of baseline at the end of 4 months. The ANA plus AFS group had a significantly higher average SFI value than the ANA group but still lower than that of the autograft group. This phenomenon was consistent with the observation that on the treadmill, the rats that performed better had less severe and fewer toe contractures, which is a known factor to interfere with SFI calculation.

In addition to the functional beneficial effects, the ANA plus AFS group also showed enhanced electrophysiological and histomorphological outcomes. The AFS cells significantly facilitated CMAP in both gastrocnemius (~0.4 E/C ratio) and tibialis anterior (~0.5 E/C ratio) muscles with electrical stimulation to the distal nerve stump near suture site. We did not find any significant differences of CMAP of the injured side between the autograft, ANA, and ANA plus AFS cell groups. However, the ANA plus AFS cell group demonstrated a lower CMAP in the contralateral uninjured gastrocnemius muscle, possibly due the prolonged experimental procedure, and might result from the side effects of isoflurane exposure [12, 17] These findings suggested that AFS cells not only accelerated the axon regeneration to a greater extent but also improved their myelination and alignment to the targeted end muscle. The electrophysiological results were in agreement with the immunohistochemical studies, which showed that the neuromuscular junction (NMJ) number was higher and the morphology of the NMJ was more complexed in the ANA plus AFS group. The efficiency and effectiveness of the regenerated nerve to reach the end muscle and replenish the motor end plates in a timely manner play a pivotal role in determining the ultimate muscle functional recovery following transection injury. In the current study, we also found that the AFS cells increased myelinated axon number and myelin sheath thickness in the regenerating nerves. Moreover, the animals from this group displayed larger myelinated axon caliber and greater *G* ratio compared with the ANA group. Along with the evidence of facilitated NMJ restoration in the end muscles, AFS cell treatment leads to beneficial outcomes in nerve regeneration, neuronal signal conduction, and muscle motor functional recovery. Thus, the results presented here have potential implications for future cell-based therapies to enhance peripheral nerve regeneration in clinical use.

To date, the solution to repair large defect peripheral nerve injuries (PNI) is limited and numerous studies have focused on designing the ideal conduits for peripheral nerve regeneration. Nerve autografts are considered the gold standard to provide the most closely native neuronal microenvironment, which preserve the Schwann cells and intact architecture of endoneurial tubes. The disadvantages of autografts are limited supply and associated donor site morbidity from additional incisions, loss of sensation, and possible painful neuroma formation [9, 27]. ANAs keep the basic extracellular (ECM) components of native nerve such as laminin and fibronectin to support and promote nerve regeneration, but the prognosis is not as satisfactory as autologous grafts as shown in the present study and other nerve transection studies [5, 28–30].

The supplement of supporting cells to the single lumen nerve conduit has been extensively researched over the past decades [31]. Schwann cell is the most important cell type in peripheral nerve regeneration in production of multiple ECM molecules and growth factors (NGF, FGF, NT-3, GDNF, VEGF, etc.) [32–34]. However, the large quantity of cells required in a limited time, slow growth rate in culture, additional surgery to harvest the cell, and the time delay from the injury all restrict the wide application of Schwann cell therapy in an acute nerve injury setting [35]. In recent years, a large body of evidence has established that stem cell augmentation of ANA could be a potentially promising alternative to provide an “off the shelf” replacement for nerve isograft [12, 22, 36, 37]. Different types of stem cells have been assessed to supplement various nerve conduits to repair peripheral nerve defects including bone marrow, adipose tissue, olfactory neuroepithelium, dental pulp, hair follicle, and dermis [11, 22, 36]. The adult stem cells from these tissues are considered multipotent but often require invasive procedure to isolate from where they reside. Fetal stem cells include embryonic stem cells (ESCs), amniotic fluid mesenchymal stem cells (AFMSC), and amniotic fluid-derived stem cells (AFS). ESCs have the greatest regenerating potential but its usage has been limited by the ethical and regulatory concerns [11]. AFS cells are a mixture of cells within the amniotic fluid from all three germ layers; these cells are demonstrated to have the capability of differentiating to various human cell types including the neurogenic cell lineage.

In this study, we have shown that the transplanted AFS cells have beneficial effects in enhancing regeneration of damaged nerve tissue, but the exact mechanisms that are responsible for these therapeutic advantages are still unknown. Several studies in rats and humans suggested that the improved nerve regeneration with AFS cell treatment are through paracrine effects [12, 15, 18, 22], which is the most likely case in the current study. The transplanted undifferentiated AFS cells participate in nerve regeneration by secreting multiple neurotrophic factors to attract and facilitate early Schwann cell recruitment to the injury site, without differentiating to Schwann cell themselves [12]. In the current study, we did not identify the fate or phenotype of these AFS cells at the end of 4 months, which warrants further investigation of the precise mechanisms and efficacy of these cells after transplantation over time. The concomitant study of tracking the AFS cells on the regenerating nerve and their possible mechanisms in facilitating nerve repair is currently underway in our lab.

In conclusion, this study has shown beneficial functional, electrophysiological, and histological outcomes in AFS cell-treated animals after sciatic nerve transection and repair. Thus, these cells may be a suitable cell source to replace Schwann cells to support and accelerate peripheral nerve regeneration flowing large gap nerve injury.

## Figures and Tables

**Table 1:** Summary of functional and histological outcomes in 3 experimental groups. ^∗∗^*p* < 0.01 and ^∗^*p* < 0.05, significantly different to the ANA group.

Functional and histological outcomes			
	Autograft	ANA	ANA+AFS
Stance/swing ratio	0.66 ± 0.22	0.64 ± 0.23	0.66 ± 0.22
Ataxia coefficient	1.06 ± 0.29	1.27 ± 0.30	1.35 ± 0.23^∗^
Overlap distance	0.79 ± 0.34	1.11 ± 0.19	0.71 ± 0.33^∗∗^
Step angle degree	0.90 ± 0.33	0.98 ± 0.37	0.97 ± 0.36
Paw angle degree	2.01 ± 0.25	2.88 ± 0.36	2.09 ± 0.22^∗∗^
Stride length	1.10 ± 0.19	1.18 ± 0.28	1.16 ± 0.14
Paw drag	1.38 ± 0.30	1.23 ± 0.38	1.08 ± 0.31^∗^
Stance width	1.41 ± 0.28	1.89 ± 0.33	1.20 ± 0.21^∗^
Axis distance	1.58 ± 0.25	1.13 ± 0.36	1.35 ± 0.23^∗^
Midline distance	1.00 ± 0.22	1.25 ± 0.27	0.92 ± 0.17^∗∗^
SFI	9.02 ± 0.63	5.41 ± 0.63	7.29 ± 0.55^∗^
Wet muscle mass ratio (gastrocnemius muscle)	0.52 ± 0.02	0.50 ± 0.01	0.51 ± 0.05
Gastrocnemius CMAP ratio	0.29 ± 0.05	0.27 ± 0.04	0.39 ± 0.05
Myelin thickness (*μ*m)	1.14 ± 0.22	0.69 ± 0.09	0.88 ± 0.13^∗∗^
Axon diameter (*μ*m)	2.29 ± 0.28	1.96 ± 0.24	2.36 ± 0.36^∗∗^
Fiber diameter (*μ*m)	3.93 ± 0.28	2.86 ± 0.25	3.84 ± 0.30^∗∗^
*G* ratio (AD/FD)	0.58 ± 0.02	0.68 ± 0.02	0.61 ± 0.01^∗∗^

## Data Availability

The in vitro and in vivo data used to support the findings of this study are included within the article.

## Additional Points

*Impact Statement*. Most previous clinical interventions for peripheral nerve injury have focused on bridging the transected axon stumps but have not incorporated stem cell therapy. In this study, a rat model was used to determine whether AFS cell plus ANA treatment accelerates nerve regeneration, reserves postsynaptic NMJ volume, and improves compound muscle action potential following peripheral nerve injury and repair. The results from this study provided evidence for future larger scale translational study and facilitate shaping the future clinical directions of AFS cell therapy for recovery after nerve injury.
